# The effects of prehabilitation versus usual care to reduce postoperative complications in high-risk patients with colorectal cancer or dysplasia scheduled for elective colorectal resection: study protocol of a randomized controlled trial

**DOI:** 10.1186/s12876-018-0754-6

**Published:** 2018-02-21

**Authors:** Annefleur E. M. Berkel, Bart C. Bongers, Marie-Janne S. van Kamp, Hayke Kotte, Paul Weltevreden, Frans H. C. de Jongh, Michiel M. M. Eijsvogel, A. N. Machteld Wymenga, Marloes Bigirwamungu-Bargeman, Job van der Palen, Marc J. van Det, Nico L. U. van Meeteren, Joost M. Klaase

**Affiliations:** 1 Department of Surgery, Medisch Spectrum Twente, PO Box 50 000, 7500 KA Enschede, The Netherlands; 2 Department of Epidemiology, Faculty of Health, Medicine and Life Sciences, Care and Public Health Research Institute (CAPHRI), Maastricht University, PO Box 616, 6200 MD Maastricht, The Netherlands; 3 Physical therapy practice, Fysio Twente, J.J. van Deinselaan 34a, 7541 PE Enschede, The Netherlands; 4 Physical therapy practice, FITclinic, Roomweg 180, 7523 BT Enschede, The Netherlands; 5 Department of Pulmonology, Medisch Spectrum Twente, PO Box 50 000, 7500 KA Enschede, The Netherlands; 6 Department of Internal medicine, Medisch Spectrum Twente, PO Box 50 000, 7500 KA Enschede, The Netherlands; 7 Department of Gastroenterology and Hepatology, Medisch Spectrum Twente, PO Box 50 000, 7500 KA Enschede, The Netherlands; 8 Epidemiology, Medisch Spectrum Twente, PO Box 50 000, 7500 KA Enschede, The Netherlands; 9 Department of Surgery, Ziekenhuisgroep Twente, PO Box 7600, 7600 SZ Almelo, The Netherlands; 10 Top Sector Life Sciences and Health (Health~Holland), Laan van Nieuw Oost-Indië 334, 2593 CE The Hague, The Netherlands

**Keywords:** Colorectal surgery, Cardiorespiratory fitness, Ventilatory anaerobic threshold, Prehabilitation, Physical therapy, Exercise training, Postoperative complications, Physical functioning/fitness

## Abstract

**Background:**

Of all older patients that opt for elective colorectal surgery, approximately one-third has one or more postoperative complications, particularly those patients with a low cardiorespiratory fitness (ventilatory anaerobic threshold (VAT) < 11 mL/kg/min). A physical exercise training program prior to surgery (prehabilitation) can improve their cardiorespiratory fitness. It remains to be seen whether prehabilitation also reduces postoperative complications, as most of the studies so far were rather underpowered, heterogeneous, and biased toward selection of patients with a lower risk of postoperative complications. The primary objective of this study is to evaluate the effects of a three-week prehabilitation program on 30-day postoperative complications in patients with a VAT < 11 mL/kg/min planned for elective colorectal resection for colorectal cancer or dysplasia.

**Methods:**

In this multicenter prospective randomized controlled trial, patients ≥ 60 years with colorectal cancer or dysplasia grade I, II, or III, planned for elective colorectal resection in two hospitals in the Netherlands, will be recruited. Eligible patients must have a score ≤ 7 metabolic equivalents on the veterans-specific activity questionnaire, and should be able to perform a cardiopulmonary exercise test. A total of 86 patients will be randomized (block-stratified randomization) to prehabilitation (intervention group) or usual care (control group). For final inclusion, VAT should be < 11 mL/kg/min. Three times a week for 3 weeks, a 60-min supervised prehabilitation session will be completed in community physical therapy practices by the 43 patients in the prehabilitation group, consisting of moderate-to-high intensity interval training to improve cardiorespiratory fitness, and resistance training to improve peripheral muscle strength. Additionally, patients perform home exercises twice a week on a moderate intensity level. The 43 patients in the usual care group will receive usual care.

**Discussion:**

Optimizing preoperative physical fitness may decrease the postoperative complication rate, may lead to fewer reoperations, less intense clinical care, a shorter length of stay, a more effective surgical planning (process-optimization), fewer readmissions, less intense rehabilitation, shorter rehabilitation period, earlier resumption of work, enhance patient perceived health-related quality of life, and promote performance in daily life. Cost-effectiveness should therefore be expected and evaluated.

**Trial registration:**

Medical Ethics Committee Twente, Enschede, the Netherlands (NL45001.044.13, September 3, 2013); Netherlands Trial Register (NTR; NTR4032, June 14, 2013).

## Background

Worldwide, colorectal cancer is the third most common type of cancer for men and women [1]. In the Netherlands, about 15.000 people are diagnosed with colorectal cancer each year [2]. Surgery is the cornerstone of treatment in patients with colorectal cancer. However, the 30-day complication rate after elective colorectal resection is approximately one-third [3], including for example an anastomotic leakage, ileus, or wound infection. Colorectal cancer primarily occurs in the elderly. The level of psychophysiological reserve capacity (resilience) and comorbidities affect the tolerance to surgery in older patients [4]. According to the Dutch Surgical Colorectal Audit, approximately 35% of the patients with colorectal cancer are aged between 60 and 70 years, and 40-50% of the patients are aged over 70 years [5]. These older patients more often have a low cardiorespiratory fitness.

In the literature, preoperative cardiorespiratory fitness has consistently been reported to be associated with postoperative outcome in major elective intra-abdominal surgery (e.g., morbidity, mortality, and length of stay) [6]. Cardiorespiratory fitness can be measured objectively using a cardiopulmonary exercise test (CPET) by determining the ventilatory anaerobic threshold (VAT). The VAT is an accurate and repeatable measurement [7] that can be obtained from the CPET, without a learning effect [8]. It is a physiological construct that occurs at submaximal exercise intensity. Hence, the VAT is only marginally influenced by the patient’s ability and motivation to deliver a maximal effort. Previous studies showed that there is a significant association between a low VAT and postoperative complications in major non-cardiac surgery [8–21]. Patients with a VAT < 11 mL/kg/min have a higher likelihood for postoperative complications [17, 22].

The literature shows that high-risk patients (those patients with a higher likelihood of postoperative complications), who participated in a physical exercise training program prior to elective surgery (prehabilitation), improved their cardiorespiratory fitness [23–25], which has led to the hypothesis that postoperative complications can be reduced by prehabilitation [23, 25–27]. Dunne et al. [28] designed a four-week high-intensity interval training program based on the work rate at the VAT for patients prior to hepatic resection. Their aim was to assess feasibility and to improve the preoperative VAT by 1.5 mL/kg/min (a 10% increase). They found their program to be feasible in sedentary healthy volunteers, resulting in a > 10% improvement in cardiorespiratory fitness (VAT). Using preoperative risk stratification, a 10% improvement in cardiorespiratory fitness in patients undergoing hepatic resection would shift 30% of the high-risk patient group to a low-risk patient group [28].

Prehabilitation programs may have beneficial effects on postoperative outcome; however, the effects on physical functioning, morbidity, mortality, and length of stay are inconclusive. In a systematic review of Moran et al. [6] that aimed to assess the ability of prehabilitation to influence postoperative outcome after intra-abdominal surgery, it was concluded that prehabilitation appears to be beneficial in decreasing the incidence of postoperative complications. Additionally, a systematic review of Bruns et al. [29] reported that prehabilitation can improve the physical fitness of older patients undergoing colorectal surgery; however, no significant effect on the reduction of complications or length of stay could be demonstrated. They concluded that previous studies on the effects of prehabilitation in patients undergoing elective colorectal resection provided no clear and complete description of the content and execution of the prehabilitation program, and that in most of these studies no adequate sample size calculation was performed [29]. Hijazi et al. [30] recently performed a systematic review of all comparative studies on prehabilitation versus usual care in patients undergoing abdominal cancer surgery. No significant difference was found in postoperative complications between prehabilitation and usual care groups. They concluded that prehabilitation programs for patients undergoing major abdominal cancer surgery remain heterogeneous in terms of their composition, duration, mode of administration, compliance, and outcomes measures used to quantify their impact. Therefore, the findings and recommendations are limited. They recommended for future research to standardize these aspects prior to the evaluation of the effects of prehabilitation programs on a larger scale [30]. Thus, it remains to be demonstrated whether a prehabilitation program reduces postoperative complications [31]. Previous studies are biased toward selection of patients with low risk of postoperative complications, and it is unlikely that a prehabilitation program of a few weeks will give a clinically relevant improvement of cardiorespiratory fitness in these low-risk patients.

In summary, current studies addressing the effects of prehabilitation on overall postoperative complications in patients with colorectal cancer are inconclusive, opposing, and of low-to-moderate methodological quality and/or therapeutic validity (concept of Hoogeboom et al. [32]). More high-quality studies are needed to validate its use in the preoperative setting [6, 33]. Recently though, it has been demonstrated that personalized prehabilitation reduced the number of patients with postoperative complications by 51% in high-risk patients undergoing elective major abdominal surgery [24]. Nevertheless, the selection of high-risk patients in this study of Barberan-Garcia et al. [24] was not performed by the use of an objective test, such as a preoperative CPET. Based on the previous published work, the current study will be different in several ways: 1) we perform a validated procedure of preoperative risk stratification with each eligible patient, in order to include only patients with a higher risk for postoperative complications based on cardiorespiratory fitness, 2) we preoperatively measure cardiorespiratory fitness objectively by using an evidence-based CPET to assess the VAT for risk stratification, 3) we design a personalized prehabilitation program based on the results of the CPET, 4) we train at a physical therapy practice close to the patient’s home and not at the outpatient clinic of the hospital, as high-risk patients and their informal caregivers are less or even unable and/or less motivated to visit the hospital to train three times a week during the preoperative period, 5) we give an exact description of the content and execution of the prehabilitation program, 6) we select a patient group with a homogeneous diagnosis, that is patients with colorectal cancer or dysplasia only, and 7) we performed an adequate sample size calculation based on previous data on the effect of prehabilitation in patients undergoing elective hepatic resection [25, 28], as well as on the reported complication rates in frail patients undergoing colorectal resection presented by Robinson et al. [34].

The primary objective of this randomized controlled trial is to evaluate the hypothesis that a three-week prehabilitation program (intervention group) will reduce the number of postoperative complications from 50% to 20% in patients with a preoperative VAT < 11 mL/kg/min who will undergo elective colorectal resection for colorectal cancer or dysplasia grade I, II, or III, when compared to usual care (control group).

The secondary objectives are 1) to observe whether changes in preoperative cardiorespiratory fitness occur in the intervention group (prehabilitation, single-arm study design), 2) to evaluate the effect of the prehabilitation program on length of stay, 3) to examine whether patients with a preoperative VAT ≥ 11 mL/kg/min, and who will therefore not be included in the randomized controlled trial, have fewer postoperative complications than patients in the prehabilitation or usual care group (for the prehabilitation group, we will use the post-prehabilitation CPET data), 4) to investigate the value of a limited geriatric assessment in this patient group to perform preoperative risk stratification, and 5) to evaluate the cost-effectiveness of prehabilitation in high-risk patients by performing a cost-effectiveness analysis.

## Methods

### Study design

This study is a multicenter prospective randomized controlled trial. The trial has started in February 2014 and will run till patient inclusion is completed (probably at the end of 2018) at Medisch Spectrum Twente in Enschede and Ziekenhuisgroep Twente in Almelo, two large community teaching hospitals in the eastern part of the Netherlands. In this manuscript, the latest version of the study protocol (version 9, March 2017) is presented. The study is approved by the Medical Ethics Committee Twente in Enschede, the Netherlands (registration number P13-18), and is registered in the Netherlands Trial Register (NTR4032). Protocol amendments will be agreed and approved by the Medical Ethics Committee Twente.

### Participants

To be eligible to participate in this study, a patient must meet all of the following inclusion criteria: 1) ≥ 60 years, 2) colorectal cancer or premalignant colorectal lesions (dysplasia grade I, II, or III) requiring colorectal resection, 3) undergoing elective colorectal resection at Medisch Spectrum Twente, Enschede, or at Ziekenhuisgroep Twente, Almelo, 4) having a life expectancy of more than 6 months as estimated by the surgeon, 5) has given informed consent to participate in this study, 6) a metabolic equivalents of task (MET) score ≤ 7 on the veterans-specific activity questionnaire (VSAQ), 7) able to perform a CPET, 8) a VAT < 11 mL/kg/min as measured at the baseline CPET, and 9) willing to perform prehabilitation at a community physical therapy practice in the adherence area of both hospitals. All patients not meeting these criteria will not be considered for inclusion. Participants can leave the study at any time for any reason if they wish to do so, without any consequences. The investigator can decide to withdraw a participant from the study for urgent medical reasons.

### Recruitment

All patients will be identified at the multi-disciplinary oncology meetings, and will be evaluated at the outpatient clinic by the surgeon or surgical resident. As most postoperative complications are to be expected in patients with a low cardiorespiratory fitness [6], we aim to include only patients who are expected to be unfit in the pre-study assessments. To select patients with a potentially low cardiorespiratory fitness, all patients identified at the multi-disciplinary oncology meeting are asked to fill out the VSAQ [35]. The VSAQ links physical activities to a particular MET score, based on the Compendium of Physical Activities [36]. Snowden et al. [14] found that patients with a VSAQ score > 7 METs are most likely not to have major postoperative complications. Therefore, we define a low perceived cardiorespiratory fitness as a VSAQ score ≤ 7 METs.

**Fig. 1:**
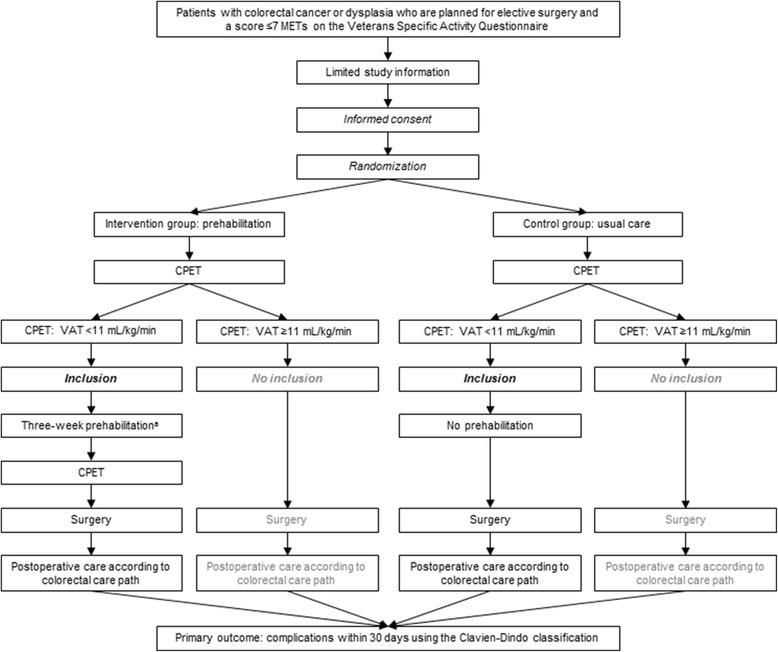
Flow diagram of the study design. ^a^: Patients with colon cancer or dysplasia grade I, II, or III will participate in the prehabilitation program in the time between contemplation of surgery and the procedure, whereas patients with rectal cancer, in case neoadjuvant therapy is needed, will complete the prehabilitation program *prior to* radiotherapy (in case of neoadjuvant radiotherapy, 5 × 5 Gy) or in the twelve-week period *after* neoadjuvant chemoradiotherapy (in week 10-12). Abbreviations: CPET, cardiopulmonary exercise test; MET, metabolic equivalent of task; VAT, ventilatory anaerobic threshold

Patients with a VSAQ score ≤ 7 METs will receive very limited information about the study. They will be asked to participate in a study addressing the role of cardiorespiratory fitness (assessed with a CPET) on postoperative complications following colorectal resection. If informed consent is obtained (Additional file 1), the patient will be randomized to the intervention group (prehabilitation) or the control group (usual care), and they will undergo a CPET to obtain objective information about their cardiorespiratory fitness, as standard work-up. Eventually, patients with a VAT < 11 mL/kg/min can participate in the study, whereas randomized patients with a VAT ≥ 11 mL/kg/min cannot participate in the study (in the intervention group, nor in the usual care group). See Fig. 1 for a flow diagram of the study design.

Patients in the prehabilitation group and the usual care group will be informed differently. The patients in the prehabilitation group will be fully informed about the study in a patient information letter that explains the aims and expectations of this study, as well as the risks and benefits of participating. Patients in the usual care group will not receive information about the possible effects of prehabilitation to avoid the risk that these patients will initiate preoperative physical exercise training themselves. The usual care group will receive a patient information letter about the registration of perioperative data and the hypothesized relation of cardiorespiratory fitness with postoperative complications after colorectal resection. Patients in both the prehabilitation group and the usual care group will be contacted by the clinical research coordinator a few days later to answer their questions, and to inform them about the CPET date. This procedure is approved by the Medical Ethics Committee Twente.

### Randomization

To analyze the effect of a three-week prehabilitation program on postoperative complications, patients with a VSAQ score ≤ 7 will be randomized in the intervention group (prehabilitation) or the control group (usual care) by using block-stratified randomization [37]. Randomization is stratified by disease and treatment type: 1) patients with colon cancer, 2) patients with rectal cancer who will receive one week of neoadjuvant radiotherapy, and 3) patients with rectal cancer who will receive five weeks of neoadjuvant chemoradiotherapy. The clinical research coordinator of the surgical department will be responsible for the randomization. Participants, care providers, and outcome assessors will not be blinded after randomization. However, the data analysts will be blinded to group allocation and are independent to the intervention. After randomization, all participants will perform a CPET for final inclusion, as merely patients with a VAT < 11 mL/kg/min can participate in the study.

### Interventions

#### Prehabilitation program

Patients in the prehabilitation group will participate in a three-week (three sessions per week, nine sessions in total) supervised physical exercise training program prior to colorectal resection. The prehabilitation program is based on the program developed by Dunne et al. [25, 28], which has been shown to be feasible in sedentary healthy volunteers [28], and later demonstrated to deliver improvements in CPET scores and perceived health-related quality of life prior to hepatic resection [25]. In the studies of Dunne et al. [25, 28] prehabilitation consisted of twelve interval training sessions over a four-week period, whereas in the present study patients will perform nine preoperative sessions of individually-tailored interval training (personalized prehabilitation) over a three-week period. In the literature, it has been shown that as little as six sessions of ‘all-out’ high-intensity interval training over two weeks is sufficient to enhance cardiorespiratory fitness and improve skeletal muscle oxidative capacity in untrained and recreationally active individuals [38, 39]. However, a comparison with our patients with colorectal cancer and a low cardiorespiratory fitness cannot be made, because their ‘all-out’ exercise program differs from our exercise intervention, as ‘all-out’ exercise may not be safe, tolerable, or appealing for our patient group.

Patients will prehabilitate on Monday, Wednesday, and Friday, or on Tuesday, Thursday, and Saturday to improve their physical fitness preoperatively. Each 60-minute training session consists of moderate-to-high intensity interval training to improve cardiorespiratory fitness, and resistance training to improve peripheral muscle strength (see Tables 1 and 2). The interval training program will be tailored to each individual patient, based on the results of the CPET. The training sessions will be supervised by a selected group of trained physical therapists in community physical therapy practices in de adherence area of the hospitals. Additionally, the patient performs unsupervised home exercises at a moderate exercise intensity (e.g., walking, cycling, or stair climbing) twice a week for at least 30 minute, as checked by the physical therapist. We added these functional exercises to the prehabilitation program as de Vreede et al. [40] demonstrated that functional-task exercises are effective at improving functional task performance in healthy elderly women. Hence, home-based functional exercises that are of relevance for a patient may have an important role in helping patients to mobilize quickly postoperatively, to be physically active throughout the hospitalization period postoperatively, and to maintain independent physical functioning. Indeed, prehabilitation ideally should not focus merely on the preoperative period. Postoperatively, hospital culture and infrastructure should stimulate the patient to be physically active. However, in most hospitals health care is entirely organized around the patient’s bed [41], which invites patients to lie in bed, even without a medical reason. Bed-centered care probably also hampers the effectiveness of prehabilitation to improve postoperative outcomes.

**Table 1:** Content of the three-week interval training program

	Duration	Intensity^a^
Warm-up	7 min	50%
Interval session	30 min	Work interval: 120% Recovery interval: 50%
Week 1, session 1, 2, and 3	Work interval: 120 s Recovery interval: 180 s	
Week 2, session 4, 5, and 6	Work interval: 140 s Recovery interval: 160 s	
Week 3, session 7, 8, and 9	Work interval: 160 s Recovery interval 140 s	
Cool-down	3 min	60%

^a^: Expressed as a percentage of the individually achieved work rate (in Watt) at the ventilatory anaerobic threshold obtained from the cardiopulmonary exercise test

**Table 2:** Content of the three-week peripheral resistance training program^a^

	Repetitions^b^	Duration	Intensity^c^
Week 1, session 1, 2, and 3	3 × 8 repetitions	20 min	70% of 1RM
Week 2, session 4, 5, and 6	3 × 8 repetitions	20 min	76% of 1RM
Week 3, session 7, 8, and 9	3 × 8 repetitions	20 min	82% of 1RM

^a^: Peripheral resistance training of the large muscle groups of the lower and upper extremities using open and closed kinetic chain exercises (without physical therapy equipment or machines): crouching by means of squat exercises (primary muscle group: quadriceps femoris; secondary muscle groups: gluteal muscles, hamstring muscles, and gastrocnemius muscle), pulling by means of pulley exercises (primary muscle groups: latissimus dorsi muscle and rhomboid muscles; secondary muscle groups: biceps brachii muscle, rotator cuff, and trapezius muscle), pushing by means of pulley exercises (primary muscle group: pectoral muscles; secondary muscle groups: shoulder muscles, triceps brachii muscle), and lifting by means of pulley exercises (primary muscle groups: trunk muscles and shoulder muscles; secondary muscle groups: quadriceps femoris, gluteal muscles, and trapezius muscle)

^b^: Rest intervals of 60-90 s between each set of repetitions

^c^: Based on the 10RM, the patient’s 1RM was determined using the Oddvar Holten diagram

Abbreviations: 1RM, one-repetition maximum; 10RM, ten-repetition maximum

Patients with colon cancer or dysplasia will participate in the prehabilitation program in the time between contemplation of surgery and the procedure, whereas patients with rectal cancer, in case neoadjuvant therapy is needed, will complete the physical exercise training program *prior to* radiotherapy (in case of neoadjuvant radiotherapy, 5 × 5 Gy) or in the twelve-week period *after* neoadjuvant chemoradiotherapy (training in the last three weeks before surgery).

#### Usual care group

Patients in the control group receive usual care and will not participate in a prehabilitation program. In addition, no advice about physical exercise training is offered to them.

### Measurements

Both the prehabilitation group and usual care group will participate in a series of outcome measure assessments at baseline, seven days postoperatively, and 30 days postoperatively, whereas the prehabilitation group will participate in a series of outcome measure assessments after the three-week prehabilitation program as well (see Table 3).

**Table 3:** Schedule of enrolment, interventions, and assessments

STUDY PERIOD	Enrolment	Allocation	Post-allocation					Follow-up	
			Pre-prehabilitation	Prehabilitation			Post-prehabilitation		
TIMEPOINT	-t_1_	0		Week 1	Week 2	Week 3		Day 7 post-surgery	Day 30 post-surgery
ENROLMENT:									
Eligibility screening	X								
VSAQ score (METs)	X								
Informed consent	X								
Allocation		X							
CPET	X								
INTERVENTIONS:									
Intervention group (prehabilitation program)									
Usual care group									
ASSESSMENTS:									
Baseline characteristics	X								
SNAQ score (0-5)	X								
VAT on CPET (mL/kg/min)			X				X^a^		
TUG test (s)			X				X^a^		
Muscle strength test			X				X^a^		
IADL			X						X
GDS 15			X						X
EORTC QLQ-C30			X						
GFI			X						X
Brief illness perception questionnaire			X				X^a^		
EQ-5D			X				X^a^		
Postoperative complications								X	X

^a^: Only for patients in the prehabilitation group

Abbreviations: CPET, cardiopulmonary exercise test; EORTC QLQ-C30, quality of life questionnaire of the European organisation for research and treatment of cancer; EQ-5D, EuroQol 5D; GDS 15, geriatric depression scale 15; GFI, Groningen frailty indicator; IADL, instrumental activities of daily living; MET, metabolic equivalent of task; SNAQ, short nutritional assessment questionnaire; TUG, timed up-and-go; VAT, ventilatory anaerobic threshold; VSAQ, veterans-specific activity questionnaire

#### Cardiopulmonary exercise test

A CPET will be used at baseline to assess cardiorespiratory fitness, as indicated by the VAT, in order to select and thereupon advice/invite eligible patients to participate in the study. For patients in the prehabilitation group, a second CPET will be used to investigate the effect of the prehabilitation program on cardiorespiratory fitness (single-arm study design). Patients free from any absolute and/or relative exclusion criteria, as stated by the American Thoracic Society and American College of Chest Physicians position statement [42], will be able to perform the CPET. The following standardized pre-test instructions will be given to the patients: 1) consume the last (light) meal at least 2 hours before exercise testing, 2) adhere to usual use of medication, and 3) wear comfortable sporting clothes and shoes. The CPET will be performed under controlled conditions at the lung function department of both hospitals, using a calibrated electronically braked cycle ergometer (Ergoline, Ergoselect 100, Bitz, Germany at Medisch Spectrum Twente and Lode Excalibur Sport, Lode BV, Groningen, the Netherlands at Ziekenhuisgroep Twente). Seat height will be adjusted to the patient’s leg length. During the CPET, patients breathe through a facemask (Hans Rudolph, Kansas City, MO, USA) connected to a Triple V volume transducer to perform breath-by-breath measurements of oxygen uptake, carbon dioxide production, respiratory flow, and volume parameters (Oxycon Pro, Jaeger, Hoechberg, Germany). In both hospitals the same respiratory gas analysis system will be used. Before each CPET at the Medisch Spectrum Twente and each morning at the Ziekenhuisgroep Twente, flow-volume calibration (three-liter syringe) and gas calibration (ambient air and a gas mixture of 16% oxygen and 5% carbon dioxide) will be performed, and ambient conditions are set. Twelve-lead electrocardiography will be used to continuously monitor heart rate during the test. Blood pressure (SunTech Tango+, SunTech Medical, Inc., Marrisville, NC, USA) will be automatically measured every three minutes to be aware of extreme hypertension or to detect hypotension. Oxygen saturation (Nonin 9600, Nonin Medical, Inc., Plymouth, MN, USA) is continuously recorded during the test by finger pulse oximetry.

After the test protocol is explained to the patient, the CPET starts with a two-minute rest period during which baseline cardiopulmonary data are collected. The patient then starts unloaded cycling at a pedaling frequency of 60-80 rotations per minute for three minutes, where after the work rate will be increased every minute by 5 to 15 W, depending on the patient’s physical fitness level and aimed at reaching a maximal effort within eight to twelve minutes [43]. The CPET continues until the patient is unable to maintain a pedaling frequency above 60 rotations per minute due to volitional exhaustion and despite strong verbal encouragement. The CPET will be considered to be at or near the maximal level when the patient shows clinical signs of intense effort (e.g., unsteady biking, sweating, and clear unwillingness to continue exercising despite strong encouragement), when the patient is unable to maintain a pedaling frequency above 60 rotations per minute, and when at least one of the following criteria is met: a heart rate at peak exercise of > 95% of predicted (predicted peak heart rate [beats/min] = 208 – 0.7 × age [years]) or a respiratory exchange ratio at peak exercise of > 1.10. After termination of the CPET, the patient will complete a recovery phase of unloaded cycling at a low pedaling frequency for five minutes. A clear description of adverse events and early test termination will be given.

During the CPET, absolute values at peak exercise are calculated as the average value over the last 30 seconds prior to termination of the test. Peak heart rate is defined as the highest heart rate achieved during the CPET. Cardiorespiratory fitness will be assessed by determining the VAT, by evaluating the peak oxygen uptake in case the patient performed a maximal effort, and by calculating the oxygen uptake efficiency slope [44]. The VAT will be detected by using the V-slope method, which is based on analyzing the slopes of oxygen uptake and carbon dioxide production. The VAT can be determined by using computerized regression analysis of these slopes. The point at which the linear slope of the relation between the carbon dioxide production and oxygen uptake changes, is called the VAT [45]. When the V-slope is expected to be unreliable, the ventilatory equivalents will be used to determine the VAT [46]. In this ventilatory equivalents method, the VAT will be defined as the point at which the ventilatory equivalent for oxygen and the partial end-tidal oxygen tension reached a minimum and thereafter began to rise in a consistent manner, coinciding with an unchanged ventilatory equivalent for carbon dioxide and partial end-tidal carbon dioxide tension [42]. The inter-observer variability in a preoperatively measured VAT has been reported to be acceptable for experienced clinicians [7], and DeCato et al. [47] recently reported short-term repeatability for CPET parameters in healthy participants in a similar test protocol, and they found no evidence for a learning effect and no significant differences in variability related to sex, age, fitness level, and diurnal factors.

#### Muscle strength

To investigate the effect of the prehabilitation program on muscle strength, a trained physical therapist will measure handgrip strength and quadriceps strength at baseline for both groups and after three weeks of prehabilitation for the prehabilitation group.

Handgrip strength (recorded in kilograms) will be assessed using the Jamar dynamometer (Sammons Preston, Rolyon, Bolingbrook, IL, USA) [48]. Handgrip strength has shown to be an indicator of skeletal muscle mass and a predictor of the risk of postoperative complications [49, 50]. The test will be performed in upright position with the elbows stretched in a straight line downwards, starting with the dominant hand. First, the handle position, in which the patient has maximum strength, will be determined. Patients will be asked to squeeze the handle as forcefully as possible for about two seconds. Hereafter, two more measurements will be performed (three grip measurements per hand). Each measurement will be followed by a pause of 15-20 seconds. Mathiowetz [48] compared the Jamar and Rolyan hydraulic dynamometers for measuring grip strength, and showed that the Jamar and Rolyan dynamometers have acceptable concurrent validity with known weights, excellent inter-instrument reliability, and strong concurrent validity.

Quadriceps strength (recorded in Newton) will be measured with a hand-held dynamometer (MicroFET2, Hoggan Health Industries Inc., West Jordan, UT, USA), which has been shown to be a feasible, inexpensive, and portable test of the quadriceps muscle strength for use in healthy older people [51]. Patients will be seated at the examination table with their lower limbs bent over the edge (knees and hips flexed at 90°). If necessary, additional stabilization will be provided at the patients’ shoulders by a second examiner. The test will be performed three times per leg, starting with the dominant leg. The hand-held dynamometer will be placed on the anterior aspect of the tibia at the level of the malleoli. The physical therapist will give resistance in the opposite direction. The patient must hold the leg in place. The resistance will gradually be increased in six seconds till maximum resistance/strength. The measurement will be stopped when the patient moves his leg. Each measurement will be followed by a pause of 30 seconds.

#### Functional mobility

To test (changes in) functional mobility, the physical therapist will execute the timed up-and-go (TUG) test at baseline for both groups and after three weeks of training for the prehabilitation group. The TUG test, a reliable and valid test, measures the time (recorded in seconds) a patient needs to stand up from a standard arm chair (approximate seat height of 46 cm), walk three meters, turn, walk back to the chair, and sit down again [52, 53]. The patient will perform the test once before being timed, in order to become familiar with the test and therefore minimize the learning effect.

In the literature, there are a few studies that demonstrated the use of the TUG test to identify patients at risk for impaired postoperative outcome [52, 54, 55]. Huisman et al. [55] showed that the TUG test was an independent predictor of the occurrence of major complications in a onco-geriatric surgical population. In this study, the predictive value of the TUG test on postoperative outcome will be determined. The TUG test is easy to perform, cheap, and does not need special equipment and might therefore be an alternative for the CPET.

#### Nutritional status

Assessment of nutritional status using the short nutritional assessment questionnaire (SNAQ) score [56] is part of usual care for our patients undergoing colorectal resection, and is evaluated at baseline for both the intervention and control group. The SNAQ is an easy, short, valid, and reproducible questionnaire for early detection of malnutrition [56]. Patients with a SNAQ score of 2 require nutritional support, whereas patients with a SNAQ score ≥ 3 require supervision by a dietician and nutritional support. In this study the SNAQ score will be used as a baseline characteristic.

#### American Society of Anesthesiologists score

The American Society of Anesthesiologists (ASA) score is the most widely used system to describe a patient’s preoperative health status. The ASA score has five classes ranging from class I, a completely healthy patient, to class V, a moribund patient who is not expected to live 24 hours with or without surgery [57, 58]. The ASA score will be assessed at baseline for both groups.

#### World Health Organization performance scale

The World Health Organization (WHO) performance scale is a tool to assess how a patient’s disease is progressing, how it affects activities of daily life, and it helps the clinician to determine appropriate treatment and prognosis [57]. The WHO performance scale will be assessed at baseline in the prehabilitation group and in the usual care group.

#### Charlson comorbidity index

The Charlson comorbidity index [59, 60] will be scored at baseline for the prehabilitation group and usual care group. It can be a useful preoperative tool in predicting morbidity and mortality outcomes in patients with colorectal cancer [61].

#### Questionnaires

Every patient will visit an oncology nurse prior to colorectal resection, which is part of usual care. The oncology nurse will ask the patient to fill out several questionnaires: the instrumental activities of daily living (IADL) [62–64], geriatric depression scale 15 (GDS 15) [65, 66], the quality of life questionnaire of the European organization for research and treatment of cancer (EORTC QLQ-C30) [67, 68], and the Groningen frailty indicator (GFI) [69]. The IADL will investigate whether a patient experiences difficulties in carrying out instrumental activities that are essential to independent living. The GDS 15 will be used to measure perceived depressive symptoms or emotional health, and has good reliability, validity, sensitivity, and specificity for older people [70]. Patients will answer ‘yes’ or ‘no’ to fifteen questions depending on how they have felt over the past week. A score of 0-4 indicates normal mood or emotional health status, 5-9 indicates mild depression, and 10-15 indicates moderate-to-severe depression. The EORTC QLQ-C30 is a reliable and valid measure of the perceived health-related quality of life of patients diagnosed with cancer in multicultural clinical research settings [67]. The GFI is a short and easy-to-use instrument to measure frailty, which is defined as a loss of resources in several domains of functioning, which leads to a declining reserve capacity for dealing with stressors [69]. The patient will be asked to fill out the IADL, GDS 15, and GFI for the second time 30 days after surgery in order to investigate the effect of surgery on the IADL, depression, quality of life, and frailty, in both the prehabilitation and usual care group.

A limited geriatric assessment will be performed at baseline for the prehabilitation group and the usual care group, measuring comorbidity with the Charlson comorbidity index, signs of depression using the GDS 15, and functional status using the IADL. We will investigate the value of the limited geriatric assessment to predict postoperative complications. In addition, the GFI will be used in order to examine whether a screening tool can replace this limited geriatric assessment.

Illness perception will be recorded in both groups by using the brief illness perception questionnaire, a quick and easy to use questionnaire [71]. The face and content properties were found to be acceptable, and the reproducibility showed moderate-to-good reliability [71]. Illness perception is an important factor of health. Patients’ personal thoughts about the symptoms they experience can be seen as one of the psychosocial factors by which variance in physical functioning in patients can be explained [71]. The physical therapist will ask patients in the prehabilitation group to fill out the brief illness perception questionnaire again after the three-week training period, in order to investigate the effect of the prehabilitation program on illness perception.

Health status will be evaluated by using the EuroQol 5D (EQ-5D) [72]. The EQ-5D questionnaire is a short profile measure of perceived health-related quality of life, and has five domains: 1) perceived mobility, 2) self-care, 3) usual activities, 4) pain/discomfort, and 5) anxiety/depression. Each domain is scored by patients according to three levels (no problems, some problems, extreme problems), for which quality weights (to enable calculation of quality adjusted life years for use in economic evaluations) are available [73]. The EQ-5D is widely used in oncology and it has shown a reasonable degree of reliability, content validity, construct validity, and responsiveness in the majority of available studies [74]. The physical therapist will ask patients in the prehabilitation group to fill out the EQ-5D again after the three-week training period, in order to investigate the effect of the prehabilitation program on health status.

### Study outcomes

#### Primary study parameter

The primary study parameter is the number of overall postoperative complications within 30 days after surgery in the prehabilitation group and usual care group. Complications will be divided in surgical and non-surgical complications. Surgical complications will be scored as anastomotic leakage, perineal wound complication, rectal stump abscess, intra-abdominal abscess, fistula, sepsis, ileus, abdominal wound complication, intestinal necrosis, stoma complication, urological complication, bleeding, iatrogenic intestinal injury, or iatrogenic vascular injury. Non-surgical complications will be scored as cardiovascular, pulmonary, thromboembolic, renal, or neurological. Complications will be recorded and graded by using the Clavien-Dindo classification [75, 76]. Readmissions within 30 days will also be recorded.

#### Secondary study parameters

Secondary study parameters are 1) cardiorespiratory fitness, as indicated by the VAT, after the prehabilitation program in the intervention group just before surgery, 2) length of stay, 3) postoperative complications within 30 days after surgery in patients with a preoperative VAT ≥ 11 mL/kg/min and who will therefore not be included in the randomized controlled trial compared to patients in the prehabilitation or usual care group (for the prehabilitation group, we will use the post-prehabilitation CPET data), 4) the value of a limited geriatric assessment in this patient group to predict (in combination with VAT) postoperative complications, and 5) cost-effectiveness of prehabilitation.

#### Other study parameters

Preoperative factors that could be associated with postoperative complications will be recorded, these include: age, sex, body height, body mass, body mass index, nutritional status, smoking status, location and type of the tumor, presence or absence of metastases, CPET parameters other than the VAT, WHO performance score, ASA score [77], Charlson comorbidity index [59], type of surgical resection, muscle strength, and the TUG test.

### Safety

All adverse events reported spontaneously by participating patients, or observed by the investigator or his staff will be recorded.

### Data analysis

#### Sample size calculation

Nowadays, there is 33% 30-day morbidity and 3.8% 30-day mortality in patients undergoing colorectal surgery in the Netherlands [3, 78]. We expect the high-risk group (patients with a VAT < 11 mL/kg/min) to have a morbidity rate of 50%. Dunne et al. stated that a 10% improvement in cardiorespiratory fitness would move 30% of the patients from high to low operative risk [25, 28]. Robinson et al. [34] showed that 21% of the non-frail colorectal patients had a postoperative complication, versus 40% in the pre-frail group and 58% in frail patients. We hypothesized a complication rate of 20% in the prehabilitation group.

For the sample size calculation we used the computer program PS Power and Sample Size Calculations version 3.0, January 2009 (Copyright © 1997-2009 by William D. Dupont and Walton D. Plummer) [79, 80]. Using an alpha of 0.0492 (due to one interim analysis) and a power of 0.80, we need 39 patients in each group to find statistically significant results. Taking into account 10% dropout, we need 43 patients in each group.

#### Procedures for data checking and entering

Data (coded on study code) will be entered in the Statistical Package for the Social Sciences for Windows (SPSS, version 23.0, IBM, SPSS Inc., Chicago, IL, USA). All data are held in an encrypted format and will be stored in a secured locked room. All variables will be checked for the number of missing, impossible or improbable values, prior to statistical analysis. In case of improbable or impossible values, the patient’s data file will be checked by the data manager. Descriptive statistics will be calculated for all variables, and distributional assumptions will be checked. The study coordinator, chief investigator, and a blinded data analyst will have access to the final trial dataset. Due to the low risk nature of the intervention, the Medical Ethics Committee determined that a data monitoring committee is not required.

#### Statistical analysis

Nominal variables will be presented as numbers with percentages. Continuous variables will be presented as mean ± SD, or as median and interquartile range, as appropriate. Data will be presented in tables and figures. All tests will be performed on the intention to treat population, and on the per-protocol population. Data distribution will be checked with help of an epidemiologist.

For categorical variables, chi-squared tests or Fisher’s Exact tests, as appropriate, will be performed to analyze the difference between the intervention and control group. For continuous variables this will be done by independent samples t-tests or Mann-Whitney U tests, as appropriate. A repeated measurements analysis (mixed models in SPSS) will be performed to assess changes over time in continuous variables. A cost-effectiveness analysis will be performed, with the use of quality adjusted life years. Withdrawn patients will be followed by checking the patient’s data file. We will use the intention-to-treat analysis to avoid various misleading artifacts.

When half of the patients needed that are calculated (43 patients) are included, a blinded independent data analyst will perform an interim analysis with a stopping rule according to O’Brien-Fleming [81]. If this interim analysis shows a significant difference in postoperative complications between the prehabilitation group and the usual care group (at *P* < 0.0054), this data analyst will contact the Medical Ethics Committee, and debate the outcome of the interim analysis and its eventual repercussions. Thereupon this committee will advise the principle investigator of the research team (JK) to decide to stop the inclusion in case of superiority. If the interim analysis shows no or minimal difference (< 10% on overall complications), the study will be stopped due to futility. Because of the interim analysis, the final *P*-value that is considered to be significant is reduced to *P* < 0.0492.

## Discussion

Surgery is an important treatment modality in patients with colorectal cancer. Unfortunately, over 30% of patients develop a postoperative complication after elective colorectal resection [3]. Older patients, especially frail patients, with a poor cardiorespiratory fitness are more prone to postoperative complications and require specific preoperative risk stratification. Optimizing preoperative physical fitness (e.g., cardiorespiratory fitness, muscle strength, and functional mobility) through prehabilitation may improve postoperative outcomes in patients undergoing major abdominal surgery [6]. Currently, there is a lack of information in the literature about the effect of preoperative exercise training on overall postoperative complications in high-risk older patients with colorectal cancer. Most of the studies so far were rather underpowered, heterogeneous, and biased toward selection of patients with low risk of postoperative complications [6, 29, 30]. The primary aim of our current randomized controlled trial is to measure the effect of a three-week prehabilitation program on postoperative complications in patients with a poor cardiorespiratory fitness, as indicated by a VAT < 11 mL/kg/min, who will undergo elective colorectal resection for colorectal cancer or dysplasia grade I,II, or III.

Postoperative complications can extend length of hospital stay, and may lead to repeated hospital admissions, chronic ill health, and a decrease or even permanent loss in functional capacity and health-related quality of life [6]. Patients with a higher cardiorespiratory fitness might be more resilient to cope with the stress associated with major abdominal surgery, with subsequent better postoperative outcomes. Optimizing preoperative cardiorespiratory fitness may therefore not only decrease postoperative complications, but may also decrease associated hospital costs, enhance the patient’s quality of life, and promote independent functioning in daily life. Our intervention fits well with the ‘prevention of limitations to function’ view in the elderly, of the Health Council of the Netherlands [82], as well as with the recently proposed new schemes of the international classification of functioning, disability, and health (ICF) to promote functioning and health [83].

According to the Dutch guidelines, patients will ideally be operated within five weeks from time of pathologic-anatomic diagnosis, and at latest within seven weeks [3]. The period between the decision for surgery and the actual surgery is limited, and therefore the valuable available time for prehabilitation is short. Moreover, some patients experience (severe) complaints of their illness, and therefore resection cannot be postponed for several weeks. However, given the need for prehabilitation interventions to be both effective and time-efficient, Weston et al. [84] indicated that carefully designed and supervised high-intensity interval training programs might be a promising perioperative strategy for enhancing cardiorespiratory fitness within a short period.

The present study, like all studies do, bares some limitations. Firstly, merely patients in the prehabilitation group will perform a second CPET to investigate the effect of the prehabilitation program on cardiorespiratory fitness. Consequently, we are not able to investigate the changes in cardiorespiratory fitness in the preoperative waiting period in the usual care group. However, the waiting period in patients in the usual care group was kept as short as possible to prevent these patients to improve their level of fitness by organizing their own preoperative exercise training program. Moreover, a study in patients planned for elective hepatic resection for colorectal liver metastases reported no significant differences in CPET variables in the control group after a four-week waiting period [25]. Secondly, the prehabilitation program in the present study consists only of a preoperative exercise training program, while it was recently indicated that studies evaluating a prehabilitation program combined with a nutritional intervention before elective major surgery in adults are producing encouraging early results [31]. However, definitive clinical evidence of physical and nutritional prehabilitation is currently very limited [31, 85, 86]. Thirdly, up till now, patient inclusion appears to be challenging. Not all patients are able and/or willing to attend the prehabilitation program due to personal, logistical, and time limitations (e.g., restricted time availability between the first outpatient visit and surgery, living too far away from the community physical therapy practice, and/or no traveling opportunities, planned vacation prior to surgery, not interested in participation). Moreover, at the start of the present study patients in both groups were informed about the possible effects of prehabilitation. We experienced that some patients in the usual care group initiated preoperative physical exercise training themselves, possibly resulting in the risk that we will not be able to detect a difference between the prehabilitation and usual care group. Therefore, we made an amendment to the study protocol (approved in April 2016 by the Medical Ethics Committee Twente) in which patients in the prehabilitation group and the usual care group will be informed differently. The patients in the usual care group will not receive information about the possible effects of prehabilitation and their elective colorectal resection was planned at the earliest convenience. Fourthly, in the present study a geriatric assessment will be completed at baseline only. Baseline data as predictors of postoperative outcome may be confounded by the effect of the prehabilitation program. Therefore, only data from patients in the usual care group will be used to investigate the value of a limited geriatric assessment to perform preoperative risk stratification. However, the power for this secondary objective might be inadequate, as the sample size calculation was not based on this objective.

We believe that preoperative exercise training in patients ‘merely’ undergoing elective colorectal resection will be only cost-effective and sustainably implementable in usual care pathways when performed in high-risk patients (those with a low cardiorespiratory fitness, e.g., a VAT < 11 mL/kg/min). Hence, an adequate preoperative risk stratification protocol, such as the protocol used in the current study (a CPET for patients with a VSAQ score < 7 METs, of which those patients with a VAT < 11 mL/kg/min at the CPET will be eligible for and advised to participate in prehabilitation), will probably lead to a feasible sustainable implementation of the prehabilitation concept. For patients undergoing elective colorectal resection and (neo)adjuvant chemotherapy and/or radiotherapy, prehabilitation might be cost-effective in all patients. Cost-effectiveness should be expected, and therefore evaluated, in terms of, among other things, fewer postoperative complications, fewer reoperations, less intense clinical care, a shorter length of stay, a more effective surgical planning (process-optimization), fewer readmissions, less intense rehabilitation, shorter rehabilitation period, and earlier resumption of work.

The strengths of our present study are the selection of high-risk patients, the clear description of the study design and prehabilitation program, the 1:1 randomization between the prehabilitation and usual care group, the use of validated measurement instruments, and the adjustment of the physical exercise training program to the cardiorespiratory fitness of the individual patient based upon the results of the CPET. Moreover, prehabilitation will be performed in a community physical therapy practice in the catchment of the patient’s home. In this way we will try to reach the frailest patients who are not capable to visit the hospital each time.

## Additional file

Additional file 1: Patient Consent Form. (DOCX 12 kb)

## Abbreviations

ASA: American Society of Anesthesiologists
CPET: Cardiopulmonary exercise test
EORTC QLQ-C30: Quality of life questionnaire of the European organization for research and treatment of cancer
EQ-5D: EuroQol 5D
GDS 15: Geriatric Depression Scale 15
GFI: Groningen Frailty Indicator
IADL: Instrumental activities of daily living
MET: Metabolic equivalent of task
SNAQ: Short nutritional assessment questionnaire
TUG: Timed up-and-go
VAT: Ventilatory anaerobic threshold
WHO: World Health Organization
