# Supplementary material for: The effects of prehabilitation versus usual care to reduce postoperative complications in high-risk patients with colorectal cancer or dysplasia scheduled for elective colorectal resection: study protocol of a randomized controlled trial
Source: BMC Gastroenterol. 2018 Feb 21;18:29. doi: 10.1186/s12876-018-0754-6 (PMC5822670; doi:10.1186/s12876-018-0754-6)
Supplement: Supplementary file 1 — Patient Consent Form. (DOCX 12 kb) [file 12876_2018_754_MOESM1_ESM.docx]

**Appendix – Patient Consent Form**

Medical scientific research

**Registration of data and/or prehabilitation in patients with colorectal cancer or dysplasia undergoing elective colorectal resection**

I was asked to give informed consent to participate in the above mentioned medical-scientific study.

**Name**:

**Date of birth**:

- I have read the patient information form. I fully understand the information and I was able to ask questions. My questions have been answered to my satisfaction.
- I know that this research will not contribute to the detection or treatment of my disease.
- I had enough time to decide whether to participate.
- I know that participation is voluntary.
- I know that I may decide at any time not to participate after all or to withdraw from the study. I do not need to give a reason for this. My treatment will not be different.
- I give permission for my GP and treating specialists (if applicable) to be informed about my participation in this study.
- I know that some people can access my data. These people are listed in this information sheet.
- I consent to my data being used in the way and for the purpose stated in the information sheet.
- I consent to my data being stored at the research location for another 15 years after this study.

I want to participate in this study.

Name of study participant:

Signature: Date: __ / __ / __

-----------------------------------------------------------------------------------------------------------------

I hereby declare that I have fully informed this study participant about this study.

If information comes to light during the course of the study that could affect the study participant's consent, I will inform him/her of this in a timely fashion.

Name of investigator (or his/her representative):

Signature: Date: __ / __ / __

-----------------------------------------------------------------------------------------------------------------

The study participant will receive the full information sheet, together with a copy of the signed consent form.
